# Presence of Merkel cell polyomavirus DNA and large-T antigen in keratinocyte carcinomas and its correlation with immunohistochemical markers p16, p53 and ki67^⋆^

**DOI:** 10.1016/j.abd.2023.12.002

**Published:** 2024-03-29

**Authors:** T.R. Bellott, Flávio Barbosa Luz, Anna Karoline Fausto da Silva, Rafael Brandão Varella, Mayra Carrijo Rochael, Rafaela Elvira Rozza-de-Menezes, Luciana Pantaleão

**Affiliations:** a Department of Pathology, Hospital Universitário Antônio Pedro, Universidade Federal Fluminense, Niterói, RJ, Brazil; b Department of Dermatology, Hospital Universitário Antônio Pedro, Universidade Federal Fluminense, Niterói, RJ, Brazil; c Department of Microbiology and Parasitology, Instituto Biomédico, Universidade Federal Fluminense, Niterói, RJ, Brazil

**Keywords:** Immunohistochemistry, Merkel cell polyomavirus, Skin neoplasms

## Abstract

**Background:**

Merkel cell polyomavirus (MCPyV), a human polyomavirus that is unequivocally linked to merkel cell carcinoma (MCC), has been found in association with keratinocytes carcinomas (KC), especially basal cell carcinoma (BCC) and cutaneous squamous cell carcinoma (cSCC). Nevertheless, there is scarce information about the possible involvement of MCPyV in the development of KC.

**Objectives:**

To assess the presence of MCPyV DNA and Large-T Antigen (LT-Ag) via Polymerase Chain Reaction (PCR) and Immunohistochemistry (IHC) in cases of KC, and to correlate its presence with immunohistochemical markers p16, p53, and ki67, tumor type and subtype, sun-exposed location, and epidemiological data.

**Methods:**

The prevalence of MCPyV DNA, LT-Ag, and immunohistochemical markers p16, p53, and ki67 was assessed by PCR and Immunohistochemistry (IHC) in 127 cases of KC, these results were correlated with tumor type and subtype, sun-exposed location, and epidemiological data.

**Results:**

The MCPyV DNA was detected in 42.57% (43 of 101) cases by PCR, the LT-Ag was detected in 16.4% (20 of 122) of cases, p16 in 81.5% (97 of 119), p53 in 66.4% (83 of 125), ki67 in 89% (73 of 82). No correlation between MCPyV LT-Ag and DNA confronted with tumor type, subtype, location site, and immunohistochemical markers was found. A single correlation between the MCPyV LT-Ag and cSCC tumors and peri-tumoral lymphocyte cells was noted.

**Study limitations:**

Further steps need to be taken to better evaluate the MCPyV influence and its possible role in KC carcinogenesis, as the evaluation of the virus genome state, the gene sequence that encodes LT-Ag in the KC tumor cells, and in situ hybridization for viral DNA or RNA in these cells.

**Conclusions:**

Despite the frequent detection of MCPyV in KC, the data available so far does not support the hypothesis of a causal relationship between them.

## Introduction

The Merkel cell polyomavirus (MCPyV), discovered by Feng and colleagues,^1^ is a human polyomavirus (HPyV) that is unequivocally linked to a rare, aggressive, neuroendocrine carcinoma of the skin, the Merkel cell carcinoma (MCC).^2^

Keratinocyte carcinoma (KC), mainly basal cell carcinoma (BCC) and cutaneous squamous cell carcinoma (cSCC), are the most common malignancies worldwide and have numerous environmental and genetic risk factors, such as UV radiation and immunosuppression.^3^ It is plausible to believe that some viruses, such as human papillomavirus (HPV) from the beta genus, could promote cutaneous carcinogenesis by maintaining cell proliferation and allowing the persistence of these keratinocytes, leading to malignancy progression in some cSCC cases.^3^, ^4^

Since its description, MCPyV has been investigated in several benign and malignant skin lesions, and its influence and possible participation in the genesis of these lesions are still under debate.^5^ The correlation between the viral presence and proliferation and carcinogenic markers, such as p16, p53, and ki67, can enlight the pathways and general role of these viruses in some skin diseases since further studies are needed for a better understanding of the relationship between HPyV and other human malignancies.^6^, ^7^

Therefore, the present study proposes to investigate the presence of the MCPyV in KC and correlate their presence with immunohistochemical markers p16, p53, and ki67, in order to clarify and deepen the possible etiological relationship between MCPyV and these cutaneous neoplasms.

## Methods

The Ethical Research Committee of the University approved this study, and participants provided signed informed consent.

### Sample and data collection

This cross-sectional study used fresh-frozen resections collected between January 2014 and 2020. Data on patient sex, age, and ethnicity, tumor location, histopathological type, and subtype were collected through patient interviews and medical records. The surgical procedure was performed according to equivalent international standards, providing tissue material for histopathological diagnosis and molecular techniques. The samples intended for the molecular analysis were immediately frozen in RNAlater Stabilization Solution (Thermo Fisher Scientific Inc., Waltham, MA, USA) at −80 °C.

### Histopathology and immunohistochemistry

Skin fragments were sent for routine histological processing at the Division of Anatomic Pathology of the University’s Hospital. Tumor diagnoses were previously defined, with further review by an experienced dermatopathologist (MCR). Subdivision in high and low-risk histological subtypes was made, with low-risk BCC represented by the superficial and nodular, and high-risk BCC represented by the infiltrative or sclerosing, micronodular, and metatypical. Low-risk cSCC was represented by the well-differentiated, and a high-risk cSCC by the poor and moderately differentiated.

**Figure 1:**
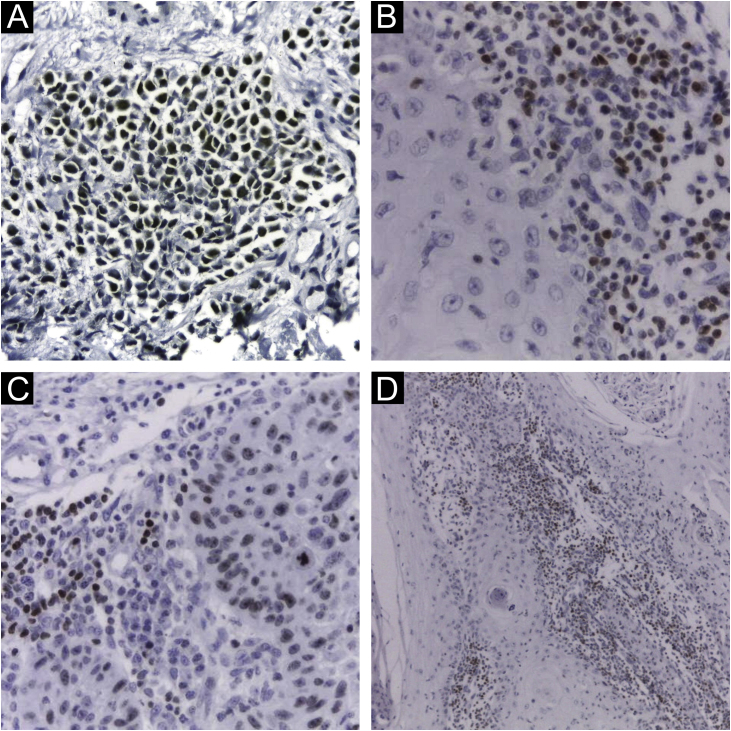
Immunohistochemistry using CM2B4 monoclonal antibody for the detection of MCPyV LT-Ag. (A) Strong staining (++) and nuclear positivity in a MCC tumor used as a positive control; (B) and (D) Strong staining (++) and nuclear positivity for cSCC peri-tumoral lymphocytic infiltrate cells; (C) Strong staining (++) and nuclear positivity for cSCC tumor and peri-tumoral lymphocytic infiltrate cells. (Plot magnification: [A, B and C] 400×, [D] 100×).

**Figure 2:**
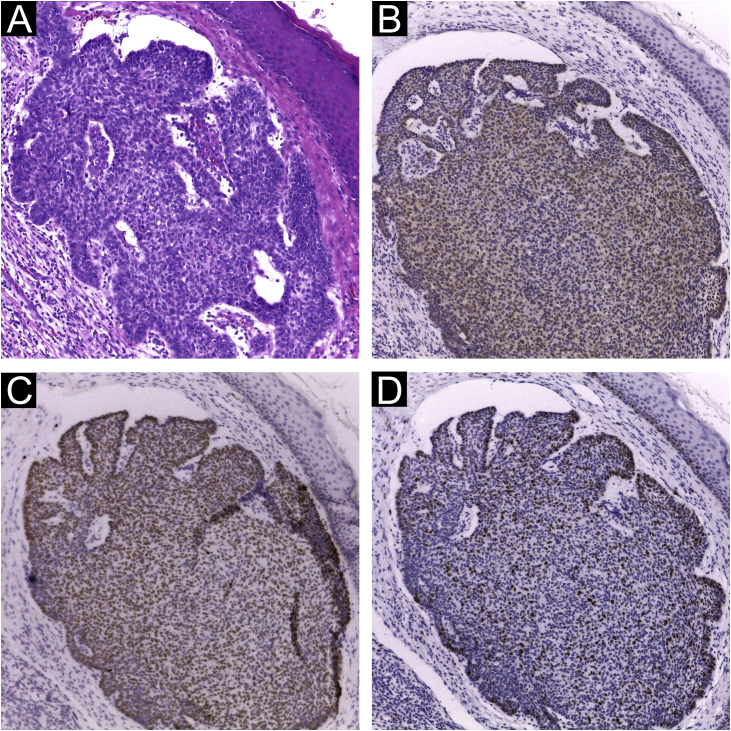
Immunohistochemical markers in a nodular BCC sample: (A) Hematoxylin & eosin stain; p16 (B), p53 (C) and ki67 (D) expression show a positive, nuclear, diffuse and strong (++) staining, marking more than 50% of the tumor cells (Plot magnification: 100×).

Immunohistochemical staining for MCPyV (Clone CM2B4, 1:100; Santa Cruz, USA), p16 (Clone JC2, 1:300, Cell Marque, USA), p53 (Clone DO-7, 1:2.000, Cell Marque, USA) and ki67 (Clone SP6, 1:300, Biocare, USA) was performed according to manufacturing instructions using HiDef Detection™️ Polymer System (Cell Marque, USA). It was documented according to the following parameters: positivity or negativity of the reaction, considered positive if any nuclear staining was identified and graded in a semiquantitative, + for weak and ++ for strong staining; localization of positivity, in the tumor or the peri-tumoral cellular infiltrate (Fig. 1). The immunohistochemical markers were also analyzed: focal or diffuse staining; marking less than or greater or equal than 50% of tumor cells; and nuclear or cytoplasmic staining. Positive controls were run in parallel (Figure 1, Figure 2).

### Detection of Merkel cell polyomavirus DNA by nested polymerase chain reaction

Approximately 10 mg of fresh frozen tissue were individually macerated using a sterile disposable scalpel and digested with proteinase K (Promega, Madison, WI, USA). DNA was subsequently extracted (RTP DNA/RNA Virus Mini Kit; Stratec Molecular Biomedical, Berlin, Germany) in accordance with the manufacturer’s instructions. Nested PCR (nPCR) for LT3 of MCPyV was performed as described previously,^8^ and bands were separated by 2% agarose gel electrophoresis, with a positive result being a clean and unique 186 bp band. All experiments were performed in triplicate, control samples were used for each genotype, and a 100 bp ladder was used as a scale (cod. 239045, Qiagen, Maryland, EUA).

### Statistics

The Statistical Package for the Social Sciences Program (IBM® SPSS® Statistics, version 29.0, USA) was used considering the 0.05 level of statistical significance. Pearson Chi-Square test or Exact Fisher test was applied to evaluate variables within MCPyV DNA and LT-Ag, correlating with participants and tumor variables using Monte Carlo simulation to estimate the p-values. The Odds Ratios (ORs) with a 95% Confidence Interval (95% CI) were also applied to estimate explanatory variables.

## Results

A total of 127 KC derived from 104 patients were available and submitted to simultaneous IHC and PCR analyses: 122 samples for MCPyV IHC, 99 for MCPyV PCR, 95 for p16, 98 for P53, and 64 for Ki67. The mean age was 70.44 years, with more than 50% of the patients having over than 70 years of age, 57 (54.8%) were male and the majority, 91 (87.5%), were caucasians. Ninety-two (72.4%) were located on the head and neck; 19 (15%) in the limbs, and 16 (12.6%) in the trunk, with 111 (85%) of them in photo-exposed areas.

### Presence of MCPyV by nested polymerase chain reaction and immunohistochemistry

In total, MCPyV LT-Ag was investigated in 122 samples and MCPyV DNA in 101 samples of KC. The cases revealed a positivity rate of 42.57% (43 of 101) in PCR and of 8.2% (10 of 122), considering as a valid result the staining only in tumor cells, associated or not with lymphocytic infiltrate cells (Fig. 1). When considering IHC as positive when marking any cells, including staining only in the peri-tumoral infiltrate cells, more 10 samples were added, with a 16.4% (20 of 122) rate of positivity. Ninety-nine samples were analyzed concurrently for MCPyV LT-Ag and DNA, with a positivity rate of 42.42% (42 of 99) in the PCR, and 8.08% (8 of 99) in the IHC (staining tumor cells, in association or not with lymphocytic infiltrate cells). The positivity for IHC doubled to 16.16% (16 of 99) when considering any staining cells (in the tumor, only in the peri-tumoral infiltrate, or both). Considering, in this study, the PCR method as the standard, only 3 of the 8 IHC-positive cases (37.5%) were also positive for PCR. Analyzing the IHC result with the inclusion of staining only in peri-tumoral cells, 8 of the 16 IHQ-positive cases (50%) had a positive correlation between the two methods. The IHC showed, respectively, a low sensitivity (7.1% and 19%) and high specificity (91.2% and 86%) compared to PCR (p < 0.001, McNemar Chi-Square test; accuracy of 56% and 58%).

### Presence of MCPyV in association with histopathological data and immunohistochemical markers

A total of 106 BCC (83.5%), 15 Cscc (11.9%), four Bowen’s disease (3.1%), and two keratoacanthomas (1.5%) were assessed. The BCC subtype and its correlation with MCPyV presence are described in Table 1, Table 2.

**Table 1:** Association between the presence of MCPyV DNA using the PCR technique and tumor type, tumor subtype, tumor location and the presence of immunohistochemical markers p16, p53 and ki67.

	PCR MCPyV			
	Negative result	Positive result	Total	p-value
**PCR MCPyV**	58 (57.43%)	43 (42.57%)	n = 101 (100%)	
**Area**			n = 101	
Non-sun-exposed	5	5	10	0.74^a^
Sun-exposed	53 (58.2%)	38 (41.8%)	91 (100%)	
	**Negative result**	**Positive result**		
**Type**			n = 95	
BCC	50 (58.8%)	35 (41.2%)	85 (100%)	1.00^a^
cSCC	6	4	10	
	**Negative result**	**Positive result**		
**BCC subtype**			n = 85	
Superficial	3	1	4	0.096^a^
Nodular	30 (52.6%)	27 (47.4%)	57 (100%)	
Infiltrative or sclerosing	15 (78.9%)	4 (21.1%)	19 (100%)	
Micronodular	1	3	4	
metatypical	1	0	1	
	**Negative result**	**Positive result**		
**BCC risk**			n = 85	
Low risk	33 (54.1%)	28 (45.9%)	61 (100%)	0.22^b^
High risk	17 (70.8%)	7 (29.2%)	24 (100%)	
	**Negative result**	**Positive result**		
**Location**			n = 101	
Head and neck	41 (53.2%)	36 (46.8%)	77 (100%)	
Trunk	5	5	10	0.068^a^
Limbs	12 (85.7%)	2 (14.3%)	14 (100%)	
**P16**			n = 95	
Negative	9	10	19	0.436^b^
Positive	46 (60.5%)	30 (39.5%)	76 (100%)	
			n = 76	
Weak	2	2	4	0.645^a^
Strong	44 (61.1%)	28 (38.9%)	72 (100%)	
			n = 76	
Focal	28 (58.3%)	20 (41.7%)	48 (100%)	0.636^b^
Diffuse	18 (64.3%)	10 (35.7%)	28 (100%)	
**P53**			n = 98	
Negative	18 (50%)	18 (50%)	36 (100%)	0.288^b^
Positive	39 (62.9%)	23 (37.1%)	62 (100%)	
			n = 62	
Weak	5	9	14	0.027^a^
Strong	34 (70.8%)	14 (29.2%)	48 (100%)	
Focal	4	4	8	0.454^a^
Diffuse	35 (64.8%)	19 (35.2%)	54 (100%)	
**Ki67**			n = 64	
Negative	7	2	9	0.464^a^
Positive	33 (60%)	22 (40%)	55 (100%)	
Weak	5	0	5	0.076^a^
Strong	28 (56%)	22 (44%)	50 (100%)	
Diffuse	31 (60%)	20 (39.2%)	51 (100%)	1.00^a^
Focal	2	2	4	

Note: Two PCR positive and two negative Bowen cases (2 of 4) and all keratoacanthoma cases positive (n = 2).

a Fisher’s exact test.

b Pearson’s Chi-Square test.

**Table 2:** Results regarding MCPyV LT-Ag research by IHC and its association with tumor type and subtype, immunohistochemical markers, and PCR.

Variable	Total (n)			
**BCC**	106 (83.5%)			
**cSCC**	15 (11.8%)			
**Bowen’s disease**	4 (3.1%)			
**Keratoacanthoma**	2 (1.6%)			
**Keratinocyte carcinoma**	127			
**Variable**	**Negative result**	**Positive result**	**Total (n)**	
**IHC MCPyV marking tumor, infiltrate and both**	102 (83.6%)	20 (16.4%)	122	
**IHC MCPyV marking tumor cells**	112 (91.8%)	10 (8.2%)	122	
**IHC P16**	22 (18.5%)	97 (81.5%)	119	
**IHC P53**	43 (34.7%)	81 (65.3%)	124	
**IHC Ki67**	9 (11%)	73 (89%)	82	
**PCR MCPyV**	58 (57.4%)	43 (42.6%)	101	
	**IHQ MCPyV marking tumor, infiltrate and both**			
	**Negative result**	**Positive result**	**Total (n)**	
**cSCC**	9 (9.3%)	6 (**31.6%**)	15 (100%)	**p-value**
**BCC**	88 (**90.7%**)	13 (68.4%)	101 (100%)	**0.017**^a^
**Superficial**	5	0	5	
**Nodular**	54 (83.08%)	11 (16.92%)	65 (100%)	p-value
**Infiltrative and sclerosing**	20 (95.5%)	2 (4.5%)	22 (100%)	0.874^a^
**Micronodular**	4	0	4	
**Metatypical**	5	0	5	
	**IHQ MCPyV marking tumor cells**			
	Negative result	Positive result	Total (n)	
**cSCC**	13 (86.7%)	2 (13.3%)	15 (100%)	p-value
**BCC**	93 (92.1%)	8 (7.9%)	101 (100%)	0.616^a^
**Superficial**	5	0	5	
**Nodular**	58 (89.2%)	7 (10.8%)	65 (100%)	p-value
**Infiltrative and sclerosing**	21 (95.5%)	1 (4.5%)	22 (100%)	0.903^a^
**Micronodular**	4	0	4	
**Metatypical**	5	0	5	

a Fisher's exact test.

The MCPyV LT-Ag detection, when including staining in the peri-tumoral infiltrate, revealed the presence of the virus approximately 4.0 times more in cSCC than BCC (40% vs. 12.8%, p = 0.017, OR = 4.513, 95% IC 1.379‒14.772). Nevertheless, no correlation was found when the IHC marked only the tumor cells, with or without the lymphocytes (Table 2).

Extensive statistical investigation was carried out to correlate the viral presence (by PCR and IHC) and the clinical and histological data provided. No correlation was found between the presence of MCPyV by PCR and IHC and the searched variables: KC types and subtypes; KC histological risk stratification; overall location; and sun-exposed sites. Also, MCPyV DNA nor LT-Ag presences were neither associated with immunohistochemical markers (Fig. 2) nor their histological features: distribution, intensity, and pattern of staining (Table 1, Table 2).

## Discussion

In this study, the presence of MCPyV was compared to a wide range of variables including KC and immunohistochemical markers, which remains a poorly investigated subject with few studies available. There was no correlation found between the MCPyV and these markers in non-MCC lesions, a similar result was viewed in a study investigating MCPyV and p16 expression in seborrheic keratosis.^9^ Having so few studies investigating the presence of cell proliferation and carcinogenesis markers in IHC, and confronting the presence of MCPyV in the same tissues, the authors had the hypothesis that a correlation between the virus and these markers could strengthen a possible etiological relationship. As there was no significant association, the result was assumed as a valid negative result, which may influence future authors to disregard this investigation.

The MCPyV is a highly prevalent virus that causes a persistent, lifelong, and usually innocuous infection in most people, and based on the Viral capsid Protein 1 (VP1) serology assay, the infection occurs as early as several months of age and increases in frequency until adulthood, when 70%–90% of all adults show evidence for persistent infection.^10^ There is a high variability of MCPyV presence by PCR in normal skin, with most data ranging from 17% to 24%,^11^, ^12^ which could explain PCR and IHC detection of the virus with no actual casual viral involvement.

The discrepancies found among viral detection by the PCR and IHC methods reinforce that there is still no gold standard for MCPyV detection, even with a multimodal method.^13^ Several conditions could explain this finding and the variability in the detection of MCPyV in KC across different studies and used methods: the PCR can be affected by the primers that were used;^14^ the quality of the samples can affect the PCR result, as several studies have reported that the detection of MCPyV DNA in fresh frozen tissues (as performed in this study) is more reliable when compared to detection in samples fixed in paraffin^15^; PCR misleading detection, since it is postulated that MCPyV is chronically eliminated through the skin, and may be part of its microbiota^12^; samples from different stages of tumor development can also justify the presence or absence of the virus, once the viral involvement could happen only in the beginning of the neoplastic process (hit and run oncogenesis)^14^, ^16^; low sensibility of the monoclonal antibody CM2B4 in tissues with fewer viral copies.^17^, ^18^

The access to only a subset of the tumor cells when extracting the PCR sample also raises the possibility that viral presence may not have been assessed, since the tumor is composed of several heterogeneous cell lineages, including immune cell infiltrates (lymphocytes, endothelial cells, and cancer-associated fibroblasts). It’s known that fibroblasts can support viral replication, acting like a genuine host cell for this virus,^19^ and also that infiltrated monocytes can be reservoirs for the virus,^20^ leading to misinterpreted PCR and IHC positive results.

Due to a lower incidence of cSCC in the general population when compared to BCC, this study had access to only 15 cases of cSCC. The result with statistical significance between the presence of LT-Ag in cSCC must be interpreted with caution due to this limited number of tumors, which impaired the statistical analyses. Also, the association only occurred when the IHC positivity was considered when staining the lymphocytic cells (combined or not with tumor cells), not persisting when LT-Ag was detected only in the tumor cells, suggesting a fortuitous correlation.

The authors considered it relevant to carry out statistical analyses of the peritumoral lymphocytic infiltrate immunostaining, with 10 of the 122 (8.2%) samples staining only the peri-tumoral infiltrate cells. Immunoreactivity in surrounding lymphocytes and stroma has unknown meaning and scarce description in the literature, and it justifies positive PCR results unrelated to infected tumor cells. IHC positivity in the peri-tumoral lymphocytic infiltrate of tumors has been rarely described^21^ and was also observed in association with mast cell staining,^22^ with no further information about the correlation with the tumor cells' IHC positivity. Low-level staining of tonsillar tissue by the CM2B4 antibody was occasionally described as well,^21^ and it was considered non-specific, not affecting the interpretation of tumor tissue staining by these authors. Studies have evaluated that the phenotypic and immunohistochemical profile of the peritumoral lymphocytic infiltrate may be related to the prognosis of cases of MCC, which, in theory, could also occur in cases of KC that had lymphocytes infected by MCPyV.^23^ At last, the immunostaining of the tumor surrounding cells is still not fully understood and has unclear significance, and the absence of additional antibodies recognizing the MCPyV also limits the result interpretation, leading the authors to consider it non-specific signaling.

Although PCR could effectively detect MCPyV DNA, it cannot distinguish the viral location, making it difficult to detach possible coincidental from causal infection. Comparatively, IHC allows the direct visualization of nuclear LT-Ag expression only in the relatively high viral load setting, which may be more indicative of causative infection.^24^

The MCC tumors reveal an interesting difference between those with and without a viral origin: as the non-viral MCC is characterized by a high tumor mutational burden, the polyomavirus-associated MCC has a low tumor mutation burden with strikingly few genomic aberrations,^2^ supporting an alternative pathway of carcinogenesis.^2^, ^25^ Hypothetically, that could also happen in KC, nevertheless, the current study did not demonstrate a correlation between MCPyV presence and sun exposure sites.

Further steps need to be taken to better evaluate the MCPyV influence and its possible role in KC carcinogenesis: state of the virus genome, whether episomal or integrated, since the viral integration seems to be a prerequisite for the development of the neoplastic process; sequence of the gene that encodes LT-Ag to determine whether there is an expression of tumor-specific truncation mutations of LT-Ag in KC; in situ hybridization for viral DNA or RNA to ensure that viral sequences are detected in tumor cells and not in cells adjacent to the tumor.^14^

## Conclusion

Further steps are still necessary to evaluate the possible etiological role of MCPyV in tumors other than MCC, however, the evidence collected until the moment still does not support this hypothesis.

## Financial support

The present study is part of the main author's doctoral thesis and was awarded a research grant from FUNADERM (Dermatology Support Fund), an organization of the Brazilian Society of Dermatology.

## Authors' contributions

Thiago Rubim Bellott: Design and planning of the study; data collection, or analysis and interpretation of data; drafting and editing of the manuscript or critical review of important intellectual content; collection, analysis, and interpretation of data; effective participation in research orientation; intellectual participation in the propaedeutic and/or therapeutic conduct of the studied cases; critical review of the literature; approval of the final version of the manuscript.

Flávio Barbosa Luz: Design and planning of the study; drafting and editing of the manuscript or critical review of important intellectual content; collection, analysis and interpretation of data; effective participation in research orientation; intellectual participation in the propaedeutic and/or therapeutic conduct of the studied cases; approval of the final version of the manuscript.

Anna Karolinne Fausto: Effective participation in research orientation; intellectual participation in the propaedeutic and/or therapeutic conduct of the studied cases; approval of the final version of the manuscript.

Rafael Brandão Varella: Effective participation in research orientation; intellectual participation in the propaedeutic and/or therapeutic conduct of the studied cases; approval of the final version of the manuscript.

Mayra Carrijo Rochael: Effective participation in research orientation; intellectual participation in the propaedeutic and/or therapeutic conduct of the studied cases; approval of the final version of the manuscript.

Rafaela Elvira Rozza-de-Menezes: Effective participation in research orientation; intellectual participation in the propaedeutic and/or therapeutic conduct of studied cases; approval of the final version of the manuscript.

Luciana Pantaleão: Effective participation in research orientation; intellectual participation in the propaedeutic and/or therapeutic conduct of the studied cases; approval of the final version of the manuscript.

## Conflicts of interest

None declared.
